# Experimental linear-optics simulation of multipartite non-locality in the ground state of a quantum Ising ring

**DOI:** 10.1038/srep07184

**Published:** 2014-11-24

**Authors:** Adeline Orieux, Joelle Boutari, Marco Barbieri, Mauro Paternostro, Paolo Mataloni

**Affiliations:** 1 Dipartimento di Fisica, Sapienza Università di Roma, Piazzale Aldo Moro, 5, I-00185 Roma, Italy; 2 Clarendon Laboratory, University of Oxford, Parks Road, Oxford, OX1 3PU, United Kingdom; 3 Centre for Theoretical Atomic, Molecular and Optical Physics, School of Mathematics and Physics, Queen's University, Belfast BT7 1NN, United Kingdom; 4 Istituto Nazionale di Ottica, Consiglio Nazionale delle Ricerche (INO-CNR), Largo Enrico Fermi, 6, I-50125 Firenze, Italy

## Abstract

Critical phenomena involve structural changes in the correlations of its constituents. Such changes can be reproduced and characterized in quantum simulators able to tackle medium-to-large-size systems. We demonstrate these concepts by engineering the ground state of a three-spin Ising ring by using a pair of entangled photons. The effect of a simulated magnetic field, leading to a critical modification of the correlations within the ring, is analysed by studying two- and three-spin entanglement. In particular, we connect the violation of a multipartite Bell inequality with the amount of tripartite entanglement in our ring.

In his 1982 paper^1^, Feynman put forward the thought-provoking idea that powerful synthetic emulations of quantum physics and chemistry would be possible by using quantum mechanical simulators rather than their classical versions. Thirty years later, the vision inherent in such a suggestive proposal could see materialisation in the form of the experimental simulation (based on light-matter interactions) of simple yet interesting quantum many-body effects^2^,^3^,^4^. Thermal states of frustrated magnets and the time evolution of spin chains of up to six qubits have been simulated in a nuclear magnetic resonance and a trapped-ion system, respectively^5^,^6^.

Due to the good level of control of the wavefunction and the high level of isolation from noise, photonics is another suitable architectures for quantum simulators^7^. Demonstrations include the design and realization of photonic settings for the quantum simulation of elementary quantum chemistry^8^, open-system dynamics^9^, and quantum walks^10^,^11^,^12^, the latter exhibiting Anderson-like disorder-induced effects. Anyonic statistics^13^ and frustration in a Heisenberg chain^14^ have been studied in analog photonic quantum simulators, while special topologically protected bound states predicted by models of condensed-matter physics have been emulated^15^,^16^.

As these examples show, faithful simulations need not implement the actual network of couplings behind the simulated many-body Hamiltonian model. Instead, one can take the approach of constructing multi-photon states enjoying the same symmetries as the ground states by using continuously tuneable quantum gates realized by means of pre-available entangled pairs of particles and measurement-induced interactions, when necessary^14^,^16^. This approach is relevant when obtaining statistical information by classical computation might be challenging, *e.g.* the spin correlation function of a one-dimensional antiferromagnetic Heisenberg model^17^,^18^. In this sense, simulating the ground state, albeit known in principle, would provide direct access to the value of such correlators.

While photonics simulation does not allow, in general, the assessment of the dynamics arising from a given Hamiltonian, it gives access to the direct engineering of interesting energy eigenstates of such models, in particular their ground state. In this paper we follow such an approach to demonstrate the nonlocal properties of the ground state of a paradigmatic many-body system: the transverse Ising model.

Close to the quantum critical point of a quantum spin model, long-range quantum correlations settle across the system as a result of the enhancement of quantum fluctuations associated with a phase transition^19^. This is manifested in peculiar behaviors of figures of merit for the quantification of general quantum correlations (from entanglement to discord), as recently shown in Refs. 20,21,22. While previous studies dealt with the quantum correlations characterising the state of two spins picked from a many-body system, Refs. 20,21,22,23 have shown that much information can be gathered from the study of global quantifiers, which are able to faithfully characterize quantum criticality even in situations where two-spin indicators fail, such as at high temperature. In order to experimentally test the occurrence of such effects in the transverse Ising model, we encode the wavefunction of a ring of three interacting spins under the effect of a simulated magnetic field using an entangled photon pair. By simulating variations of the effective magnetic field, we study the changes in the quantum correlations between the spins of the simulated model, which are encoded in multiple degrees of freedom of the photon pair. We focus, in particular, on the multipartite Bell-like inequality embodied by the Svetlichny formulation^24^. The violation of such inequality witnesses the occurrence, in the state of a system, of genuine multipartite non-local correlations. We show that the Svetlichny function evaluated using the simulated ground state of the Ising chain violates the *N*-party local-realistic bound that, even for a short chain, is very close to the quantum critical point defined in the thermodynamic limit *N* → ∞. By exploiting the analytical link between the Svetlichny parameter and the measure of genuine multipartite entanglement embodied by the three-tangle^25^ and the tripartite negativity^26^, we estimate the degree of tripartite entanglement shared by the spins of an Ising chain at a set degree of violation of the Svetlichny inequality

## Results

### The system

**Figure 1:**
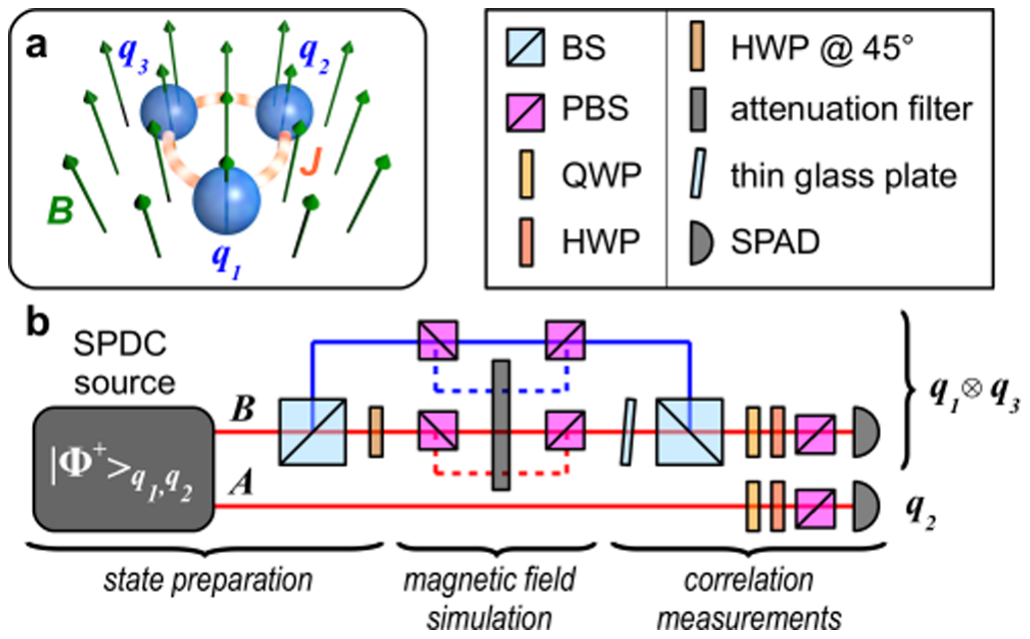
Experimental set-up. (a) Schematic picture of the three-spin ring (*q_i_*, *i* = 1, 2, 3) in a transverse magnetic field *B*. The spin-spin coupling strength is *J*. (b) Conceptual sketch of the photonic setup. SPDC source: spontaneous parametric down-conversion source generating photon pairs in the entangled state |Φ^+^〉 of spins *q*_1_ and *q*_2_. BS: beam-splitter; PBS: polarizing beam-splitter; QWP: quarter-wave plate; HWP: half-wave plate; SPAD: single photon avalanche photodiode. For photon B, the red (blue) line between the BSs corresponds to the transmitted (reflected) path (spin *q*_3_), and the solid (dashed) line between the PBSs correspond to the horizontal (vertical) polarization of photon B (spin *q*_1_).

We have realized the ground state of the Ising ring 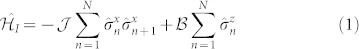
with 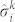 the *k* Pauli operator of spin *i* and 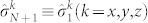. In Eq. (1), 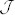 is the inter-spin coupling strength and 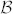 is the magnetic energy of the spins subjected to a global transverse magnetic field [cf. Fig. 1 (a)]. Besides being a key Hamiltonian in quantum statistical mechanics as it embodies one of the simplest models to show a phase transition, 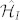 has also attracted much attention from the quantum information community in light of the interesting quantum correlation properties of its ground state^19^,^27^. As shown in Ref. 27, for 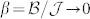, the ground state |*g_N_*(*β*)〉 of the Ising model approaches an *N*-spin state that is locally equivalent to the Greenberger-Horne-Zeilinger (GHZ) state 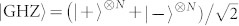 with |±〉 the eigenstates of 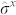, thus exhibiting long range quantum correlations and multipartite quantum entanglement. While most of the attention has been focused on the behaviour of bipartite entanglement, it has been shown in Ref. 23 that |*g*(*β*)〉 is endowed with strong multipartite non-local properties, as witnessed by the violation of the Svetlichny inequality. For *β* → 0, the degree of violation approaches the maximum allowed value of 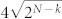 with *k* = 1 (*k* = 2) for an even (odd) number of spins, which is achieved for an *N*-spin GHZ state^28^, thus reinforcing the claim on the form of the ground state in such limit^23^. Quite remarkably, the violation of a generalised Svetlichny inequality occurs close to |*β*| = 1, which for a ring in the thermodynamic limit (*N* → ∞) at zero temperature identifies the quantum critical point of the quantum Ising model at which a ferromagnetic-to-paramagnetic phase transition occurs^29^. In this context, the total spin magnetization plays the role of an order parameter, which exhibits a singularity at the critical point. For the finite-size chain addressed here, there cannot be a direct link between the establishment of such onsets and the model's quantum phase transition. A connection, on the other hand, should be searched with the changes of symmetries in the system occurring close to the critical point, which can affect the way quantum correlations are shared by the spins.

To simulate the low-lying part of the spectrum of a system and its changes, one could make use of the adiabatic theorem: an initial Hamiltonian can be adiabatically evolved to a final one so as to induce a corresponding change in its ground state. An adiabatic quantum simulator can thus be built by engineering interactions among particles using tunable external parameters (e.g. an external magnetic field). The system will remain in its ground state if the system parameters change slowly enough. In our work we used a tuneable operation without the necessity of either discretizing the quantum evolution or engineering physical interactions. This is in line with the approaches used in Ref. 14, 16, 30. We thus consider a special form of analog simulation that tracks the ground state of the system rather than its Hamiltonian, and for which the change of quantum evolution is made through a tuneable gate. The ground state of an *N* = 3 spin Ising ring with *β* < 0 (realised considering 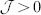 and 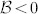) reads 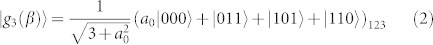
with 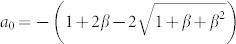. As mentioned, for *β* → 0 such state is locally equivalent to a GHZ state. We thus aimed at implementing a quantum circuit that achieves such state. We use a source of polarization-entangled photon pairs that generates, by spontaneous parametric down-conversion (SPDC), the entangled state of spins 1 and 2 
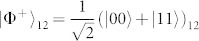
, which is encoded in the polarization state 
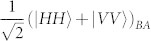
 of photons *A* and *B* [cf. Fig. 1 (b)]. Here, |*H*〉 and |*V*〉 stand for the horizontal and vertical polarization states of a photon. The third spin is encoded in the path degree of freedom of photon B by passing it through a 50-50 beam splitter (BS) and creating the state 
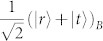
. This encodes the logical state 
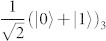
. Finally, spin 3 is entangled to spin 1 by a controlled-NOT gate that is physically implemented between the path of photon B (the control spin) and its polarization (the target one): a half-wave plate (HWP) with its axis oriented at 45° with respect to the horizontal direction and inserted in the transmitted path of photon B performs a bit-flip on spin 1 depending on the state of spin 3. We thus obtain the state 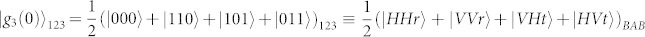 which corresponds to the ground state of the spin ring with no magnetic field and is locally equivalent to |GHZ〉_123_ upon application of the three-spin Hadamard transform 
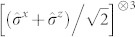
.

The changes in the transverse magnetic field resulting in *β* ≠ 0 and the possibility to explore the properties of the ground state of the Ising ring, is then simulated by introducing a variable attenuation on photon B depending on the joint state of spins 1 and 3. This is achieved by first splitting the four path-polarization components of photon B with two polarizing beam-splitters (PBS) and then inserting a variable attenuation filter on the modes corresponding to |011〉_123_, |110〉_123_ and |101〉_123_. Correlations among the spins can then be determined through coincidence measurements in different projection bases. This conceptual scheme is implemented in a doubly displaced Sagnac interferometer to guarantee the perfect phase stability of the state. The tunability of the parameters that enter our experimental simulator allowed us to achieve *β* ∈ [−2, 0], which are sufficient to explore the most salient features of the model at hand. Details on the source and on the experimental procedure are reported in the Methods.

### Entanglement characterization

Our goal is to characterise the fundamental symmetry changes occurring in the ground state of the Ising ring when crossing the quantum critical point by assessing multipartite non locality and entanglement. In order to achieve this, we have measured the amount of both bipartite and tripartite entanglement in the simulated |*g*_3_(*β*)〉 for each value of *β* in our experiment. We perform our characterisation of the quantum correlation properties of the model in a gradual manner, starting for an assessment of the bipartite entanglement between any two spins taken out of the ring. This is pursued by considering the entanglement witness operator 
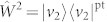
, where |*v*_2_〉 is the eigenvector of 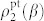 associated with the smallest eigenvalue^31^. Here, pt stands for the partial transposition operation and *ρ*_2_(*β*) is the two-spin reduced state 
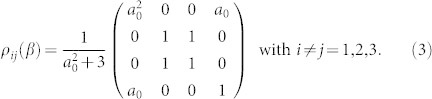
Achieving 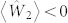 guaranties bipartite entanglement in the state of the spin pair. Although the translational invariance of the Ising ring makes any spin-pair equivalent, in our experiment we have considered spins 2 and 3 and decomposed the witness into the combination of local measurement settings 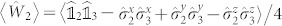. When calculated over separable states, we have 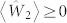, so that a negative value signals non-zero bipartite entanglement.

As it has been shown in Refs. 20–23, two-point correlation functions are in general suitable witnesses of criticality only at strictly null temperature. For non-zero tempertature, on the other hand, their structural changes occurring at criticality is quickly overcome by thermal fluctuations, rendering them ineffective to reveal the occurrence of a quantum phase transition. This is not the case as far as multi-point correlations are considered. In fact, as at criticality the correlation length of a critical system typically diverges, it is a global indicator of correlations that should be addressed. Our study gives strong experimental evidence of this feature by investigating critical structural changes in the multipartite non-locality of |*g*_3_(*β*)〉. This is done by demonstrating experimentally the violation of the tripartite Svetlichny inequality^24^, which would witness genuine tripartite non-locality in the same manner as the Bell inequality does for bipartite non-locality. The inequality is written as 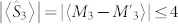 with the Svetlichny function 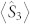 that can be built as the combination of the Mermin-Ardehali-Belinskii-Klyshko functions^28^,^32^,^33^,^34^ 〈*M*_3_〉 and 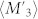 given in the Methods section. The Svetlichny inequality has been violated using a photonic GHZ state in Ref. 35. It is straightforward to show that one expression of 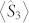 which maximizes the violation for the state at hand is 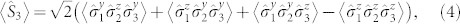
which can be easily measured in our set-up by implementing four local measurement settings. An important point should be stressed here: needless to say, being the transverse Ising model, exactly solvable, its ground state is perfectly known. However, our goal here is the assessment of a global figure of merit that would require, in principle, an optimization over a large number of parameters [such optimization his already inherent in Eq. (4)]. As the size of the ring grows, the problem would become quickly intractable and no closed formula is known to hold, currently. It is thus clear that the implementation of a quantum simulator able to assess directly the multi-point correlation function needed to study the N-party Svetlichny function 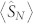 will be the only way to attack the problem. Our experiment embodies the demonstration of the viability of such an approach for the first non-trivial case of spin ring, i.e. *N* = 3.

**Figure 2:**
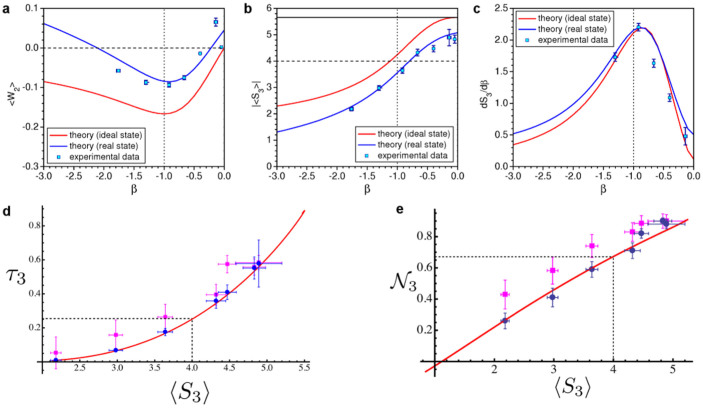
Results. Measured values of (a) the bipartite entanglement witness 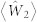, (b) the Svetlichny function 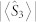 and (c) its derivative 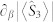 as a function of *β*. The square dots show the measurements performed on the ground state; the red line shows the expected behaviors for the ideal ground state; the blue line represents the theoretical values for a state affected by noise. The vertical dotted line identifies the ‘critical point' *β* = −1; the horizontal dashed line in (a) and (b) show the lowest (highest) possible value that can be achieved by 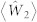 (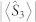) for a (bi-)separable state; and the full black horizontal line in (b) shows the highest value that can be achieved by a GHZ state. (d) Relation between 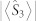 and *τ*_3_ for the ground state |*g*_3_(*β*)〉. The dashed vertical line marks the local realistic bound imposed to the Svetlichny parameter. This identifies the threshold value *τ*_3_ = 0.25 above which the state is non-local in a tripartite sense. The (blue) circle-shaped points are the values of *τ*_3_ obtained using the analytic relation with 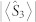 discussed in the body of the paper, evaluated at the experimental values of the Svetlichny parameter. The (magenta) square-shaped data points are the values of *τ*_3_ estimated using local measurement settings. (e) Analogous plot for 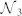. In this case, the threshold for tripartite non-locality is 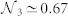. The same color-code used in panel (a) holds here. Error bars are determined by standard error propagation with Poissonian distributions attached to the experimental counts.

From the knowledge of 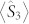 it is also possible to estimate the value of measures of genuine tripartite entanglement. For instance, for the case at scrutiny here, we can link the violation of the Svetlichny inequality to the measure of tripartite entanglement embodied by the *three-tangle* 
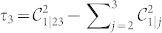
25 with 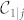 the concurrence of the bipartite state composed of spin 1 and *j* = 2, 3 and 
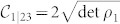
 that measures the entanglement between spin 1 and the two-spin system composed of spins 2 and 3. Another measure that can be linked to 〈*S*_3_〉 is the *tripartite negativity* 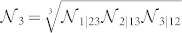 with 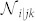 the negativity of the bipartite system composed of spin *i* and (*j*, *k*)^26^. By calculating explicitly each element of Eq. (4) for the state |*g*_3_(*β*)〉 and inverting the relation between *a*_0_ and |〈*S*_3_〉, it is straightforward to link analytically the degree of genuine tripartite entanglement as quantified by *τ*_3_ and 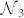 to the values taken by the Svetlichny function 〈*S*_3_〉 as 
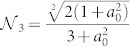
 with 
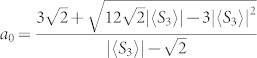
 and 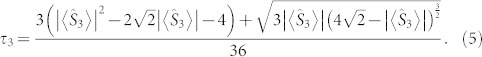
The functional link between such measures of multipartite entanglement and the Svetlichny function is shown in Fig. 2, where we have identified a threshold value of *τ*_3_ and 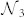 above which genuine tripartite non locality is ensured. Notice that, while the value of *τ*_3_ is strongly determined by the similarity between our resource state and a GHZ state (and is null, for instance, for a *W* state such as 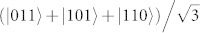), this is not the case for 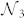, which can attain significant values also for other forms of multipartite entangled states, not necessarily of the GHZ form. The results of the measurements performed on the simulated state are presented in Fig. 2 as a function of *β*, together with the theoretical predictions. Moreover, we have been able to provide an estimate of the values taken by *τ*_3_ for the state of the simulated chain at hand made on the basis of the experimentally measured values of 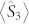 and a comparison with the value that can be reconstructed by writing both *τ*_3_ and 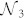 in terms of multi-spin correlation functions and putting together the outcomes of our experimental measurements (cf. Fig. 2 d). This comparison includes the experimental imperfections of our simulator, in particular we have observed some background noise whose amplitude depends on the set value of *β* (cf. Methods).

## Discussion

As seen from Fig. 2, the quantum correlations of *ρ_exp_*(*β*) undergo a profound modification at the critical point. First, the bipartite non-locality (Fig. 2 a), which is null for *β* → 0 and *β* → −∞, reaches a non-zero value close to the point of structural changes 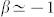, which would correspond to the critical point for *N* → ∞. At the same time, the tripartite non-locality witnessed by 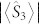 [Fig. 2 (b)], which is maximum for *β* → 0, when the ground state is close to a GHZ state, decreases as |*β*| increases and reduces to values lower than the non-locality threshold just after 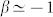. The inflexion point that is visible in the behavior of 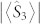 against *β*, close to the expected critical point, reveals that 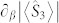 will be maximum at that point. This is clearly seen in Fig. 2 (c), which demonstrates the sensitivity of the figure of merit embodied by 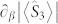 to the modifications undergone by the structure of multipartite quantum correlations for 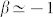.

Let us also notice that the noise affecting the simulated state only induces a quantitative reduction on the Svetlichny parameter but does not change the value of *β* at which the modifications to the sharing of quantum correlations occur. The maximum of 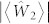 and the point of inflexion of 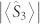 are found at 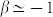 independently of the amount of noise affecting the state. This guarantees the detection of the critical point even though the system is not perfectly simulated, which is encouraging for potential medium-size systems. On the other hand, the mixed nature of the state affected by noise demands the modification of the definition of *τ*_3_ and its reformulation in terms of convex-roof extensions^25^. The calculation of *τ*_3_ for general three-qubit states is a very demanding task that goes beyond the scopes of our experimental work. However, the results in Fig. 2 (d) show that both our experimental reconstruction and the values estimated using Eq. (5) are very close to the behavior expected for the proper ground state of the simulated chain, hinting strongly at the high quality of the data. Moreover, 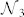 (complemented with the information on multipartite inseparability provided by the violation of the Svetlichny inequality) is well suited for three-spin mixed states without modifications to the definition above. This has enabled us to generalise the link between 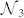 and 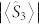 so as to provide a non-tomographic estimate of the tripartite entanglement content of *ρ_exp_*(*β*) based on the experimental values of 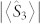. These are reported in Fig. 2 (e) (see also Table 1), showing the excellent agreement of our estimates and the expected relation between 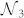 and 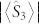.

We have experimentally studied multipartite non-locality in the ground state of an Ising ring undergoing important changes at the level of the quantum-correlation sharing. A major step forward in this context would be embodied by the simulation of non-zero temperature equilibrium states. An interesting approach to this problem has been reported in^30^. The simulation of thermal equilibrium states will pave the way to the investigation of interesting many-body effects in photonics quantum simulators^7^, from criticality to thermodynamics.

## Methods

### Experimental set-up details

**Figure 3:**
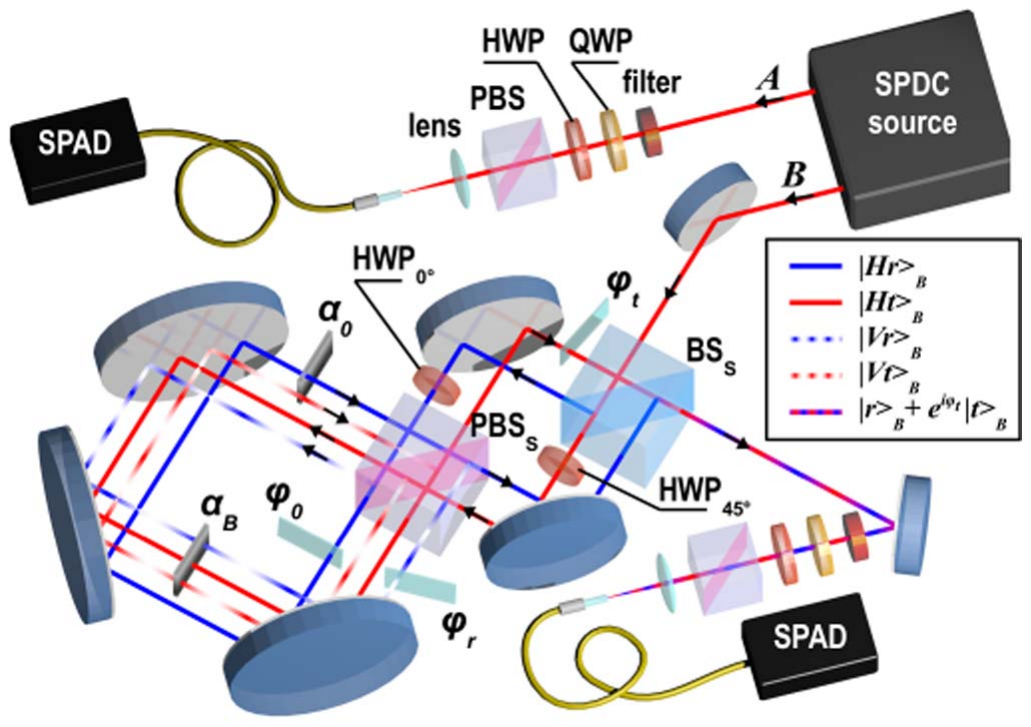
Actual experimental implementation of the photonic simulator based on a doubly displaced Sagnac interferometer setup. SPDC source: spontaneous parametric down-conversion source generating photon pairs in the entangled state |Φ^+^〉 of spins *q*_1_ and *q*_2_. BS*_S_*: beam-splitter; HWP_45°_ and HWP_0°_: 45°-oriented and 0°-oriented half-wave plates; PBS*_S_*: polarizing beam-splitter; *α_B_* and *α*_0_: attenuation filters; *ϕ_t_*, *ϕ*_0_ and *ϕ_r_*: glass plates; QWP: quarter-wave plate; HWP: half-wave plate; SPAD: single photon avalanche photodiode. For photon B, the red (blue) line inside the Sagnac interferometer corresponds to the transmitted (reflected) path (spin *q*_3_), and the solid (dashed) line after the PBS correspond to the horizontal (vertical) polarization of photon B (spin *q*_1_).

The experimental photonic quantum simulator consists in the doubly displaced Sagnac interferometer setup presented in Fig. 3 that allows to prepare the two-photon three-qubit state |*g*_3_(0)〉_123_, simulate a transverse magnetic field and measure two-photon coincidence detection events in different projection bases of the three spins. The conceptual setup was already described in the main text, in Fig. 1 (b); here we clarify the correspondence between the actual Sagnac-based setup and the former and give details on the role played by the different optical elements.

The source of polarization-entangled photon pairs we used is based on Ref. 36: it consists of a 0.5 mm-long type-I *β*–barium borate crystal (BBO), pumped back and forth by a CW laser beam at 355 nm (100 mW), and emitting photon pairs by spontaneous parametric down-conversion (SPDC). After the first passage of the pump beam in the crystal, both the pump beam and the first emission cone of photon pairs are reflected back using a spherical mirror, with a rotation of 90° of the polarization of SPDC photons. A second cone, superimposed with the first one, is emitted at the second passage of the pump beam through the crystal. This allows, by selecting two diametrally opposite spatial modes *A* and *B* of the cones, to generate the input state 
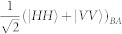
 of photons *A* and *B*. Frequency-degenerate photons are selected by two interference filters centered at 710 nm with a bandwidth of 10 nm, and detected by single photon avalanche photodiodes (SPADs) connected to single-mode optical fibers.

Typical detected coincidence counts where of the order of 100 Hz when the attenuation filters were all tuned at their maximal transmission. For all measurements reported in Figs. 2, the integration time was chosen to accumulate around 4000 coincidence events; this ranged from 60 s for |*β*| = 0 to 400 s for |*β*| = 1.

The entanglement quality of the state was partially estimated during the alignment of the set-up: the 2-qubit polarization state measured on the transmitted path (respectively on the reflected path) gave a fidelity of 92% to |Φ^+^〉 (respectively 89% to |Ψ^+^〉); the visibility of the path qubit interference in the Sagnac interferometer was measured to be 90%.

The preparation of |*g*_3_(0)〉_123_ is achieved thanks to the beam-splitter BS_S_ and the 45°-oriented half-wave plate (HWP_45°_) in the transmitted path. The optical delay introduced by this HWP was compensated by a second HWP with its axis at 0° with respect to the horizontal direction in the reflected path (HWP_0°_).

The effect of the transverse magnetic field is then simulated by splitting the polarization components of photon B, both for the reflected and transmitted path, with the polarizing beam splitter PBS_S_ and introducing a variable attenuation filter (*α_B_*) common to the paths |*Ht*〉*_B_*, |*Vr*〉*_B_* and |*Vt*〉*_B_*. Again, a second glass slab (*α*_0_) on the path |*Hr*〉*_B_* is used to maintain paths of equal optical length in the interferometer. The polarization components are then recombined on the PBS before projective measurements are performed.

Let us notice that, because of an unbalance in the BS double transmission (*η_t_*/2 = 66%) and double reflection (*η_r_*/2 = 34%) coefficients, as well as in the SPDC source HH-cone (*η_HH_*/2 = 58%) and VV-cone (*η_VV_*/2 = 42%) emission probabilities, the state that is actually generated in our experiment is 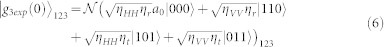
with 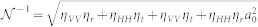. This is why we chose to trace out qubit 1 for the evaluation of the bipartite entanglement witness *W*_2_: we can see that in this case we obtain more balance between the |Φ^+^〉 and |Ψ^+^〉 parts of the reduced density matrix *ρ*_2_, as is the case in the ideal ground state. The correspondence between the attenuation coefficient and the simulated value of *a*_0_ (and thus of *β*) is given by 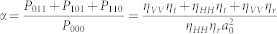, where *P_ijk_* stand for the probability of two-photon coincidence of state |*ijk*〉_1,2,3_. In the experiment, we could vary *α* from 0.1 to 4, and thus achieve values of *β* within the interval [−2; 0].

Finally, projective measurements are done on both photons before registering their coincidence detection with two single photon avalanche photodiodes (SPAD) and a coincidence counting electronics. For both polarization qubits, this is achieved by a standard polarization analysis set-up consisting of a quarter-wave plate (QWP), a HWP and a PBS, while for the path qubit the projection on different basis is achieved by the second passage through BS_S_ and a glass plate (*ϕ_t_*) that can be rotated in the transmitted path so as to change the relative phase between |*r*〉*_B_* and |*t*〉*_B_*. The optical delay introduced by this glass plate is compensated by two glass plates (*ϕ*_0_ and *ϕ_r_*) inserted in the reflected path. Note that these two glass plates are also used to correct for the *π* phase difference introduced by HWP_0°_ between the horizontal and vertical polarization components of the reflected path.

### Noise considerations

A fair comparison between experimental results and theoretical predictions will have to account for the imperfections of the optically simulated ground state with respect to the ideal one |*g*(*β*)〉. Specifically, we have observed some background noise whose amplitude depends on the set value of *β*. In particular, the real photonic state is affected by background noise that mixes the ground state of the spin ring to white noise, so to obtain the Werner state 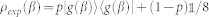, where *p* = *R_SNR_*/(2 + *R_SNR_*) depends on the signal to noise ratio *R_SNR_*. In our simulator, the value of the effective magnetic field is changed by attenuating the signal in specific paths in the interferometers. This implies that there is less signal in those paths for large values of |*β*| than for small ones, which results in a *R_SNR_* that diminishes with increasing values of |*β*|. In turn, this makes *p* dependent on *β*. For each value of |*β*|, we estimated the signal to noise ratio as: 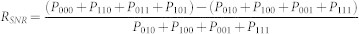, where *P_ijk_* stand for the probability of two-photon coincidence of state |*ijk*〉_1,2,3_, from which we obtained values of *p* as a function of *β*. A linear fit of these experimental values gave us *p* = 0.128*β* + 0.927, which allowed us to compute the blue theoretical curves in Fig. 2 (a), (b) and (c). The good agreement between the measured and theoretical values confirms that this simple model captures the main imperfections of our setup.

### Expression of the Svetlichny inequality

The Svetlichny inequality can be expressed as 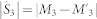 with *M*_3_ = *E*(*a*_1_, *b*_1_, *c*_2_) + *E*(*a*_1_, *b*_2_, *c*_1_) + *E*(*a*_2_, *b*_1_, *c*_1_) − *E*(*a*_2_, *b*_2_, *c*_2_) and 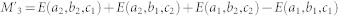
23,24,28,34 the Mermin-Ardehali-Belisnskii-Klyshko function^32^,^33^. Here, *E*(*a_i_*, *b_j_*, *c_k_*) (*i*, *j*, *k* = 1, 2) is the statistical correlation function for local spin measurements with the angle settings *a_i_*, *b_j_*, and *c_k_* respectively: for each setting we have *E*(*a_i_*, *b_j_*, *c_k_*) = *A*(*a_i_*)
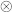
*A*(*b_j_*)
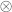
*A*(*c_k_*), with 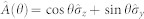. In the case of the ground state of the N = 3 Ising ring, it can be shown that 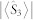 is maximal for *a*_1_ = 3*π*/4, *a*_2_ = *π*/4, *b*_1_ = *c*_1_ = *π*/4 and *b*_2_ = *c*_2_ = −*π*/4.

### Estimation of the tripartite negativity

In Table I we report the values of the tripartite negativity that could be deduced from the measured values of 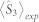.

## Author Contributions

M.B. and M.P. conceived the original idea of the simulation. A.O., J.B., M.B., and P.M. devised the experimental apparatus and performed the experiment. A.O., M.B., and M.P. analysed the data and performed the theoretical simulations. All authors contributed to the preparation of the manuscript.

## Figures and Tables

**Table 1:** Tripartite negativity. Estimates of the tripartite negativity in the noise-affected ground state of a three-spin Ising ring corresponding to the experimental values of the Svetlichny function, here dubbed 〈*S*_3_〉*_exp_*, measured at the values of *β* reported in Fig. 2

〈S_3_〉_exp_	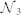
4.83 ± 0.15	0.90 ± 0.02
4.89 ± 0.31	0.88 ± 0.03
4.47 ± 0.12	0.82 ± 0.03
4.32 ± 0.13	0.71 ± 0.05
3.64 ± 0.10	0.59 ± 0.05
2.98 ± 0.09	0.41 ± 0.06
2.18 ± 0.07	0.26 ± 0.05
